# Effect of agriculture and vegetation on carbonaceous aerosol concentrations (PM2.5 and PM10) in Puszcza Borecka National Nature Reserve (Poland)

**DOI:** 10.1007/s11869-015-0378-8

**Published:** 2015-11-12

**Authors:** Agnieszka Witkowska, Anita U. Lewandowska, Dominika Saniewska, Lucyna M. Falkowska

**Affiliations:** Institute of Oceanography, University of Gdańsk, Al. Marszałka J. Piłsudskiego 46, 81-378 Gdynia, Polska

**Keywords:** PM2.5 and PM10, Organic carbon, Elemental carbon, Agriculture influence, Polish National Nature Reserve

## Abstract

Elemental carbon (EC) and organic carbon (OC) concentrations were measured in PM2.5 and PM10 samples collected at Diabla Gora (Puszcza Borecka National Nature Reserve, Poland) between 1 January and 31 December 2009, to investigate the seasonal and daily concentration variations and source regions. Strict sampling and measurement procedure, together with analysis of air mass backward trajectories and pollutant markers, indicated that the most important sources of carbon in the aerosols over Diabla Gora were vegetation, agricultural activity, and biomass burning. The highest contribution of secondary organic carbon (SOC) in aerosol mass (70 %) was detected during summer as a result of increased vegetation. In spring and autumn, raised concentrations of primary OC, calcium, and potassium and the presence of ammonium nitrate were observed in aerosols due to emission from surrounding fields and forests, as well as from fires in Lithuania. Anthropogenic influence on the increase in concentration of all carbon species was observed only in winter, when air masses drifted in from habitations situated within a radius of 50 km from the Diabla Gora station. Transport was of sporadic significance to the measured concentrations, and only in PM2.5 when wind speed was close to 1 m s^−1^. In this case, the concentration of EC rose several fold. Such a tendency was particularly noticeable with the influx of air masses from nearby cities and the Polish-Russian border, which is located 29 km away from the station.

## Introduction

Particulate matter (PM) is still a significant atmospheric pollutant in Europe (Pateraki et al. 2012). In Poland, the problem of high aerosol concentration is even more notable than in highly industrialized countries such as the UK, Germany, France, Norway, or Finland, since the most important source of pollutants is coal combustion in the communal sector (NEM 2009; EMEP 2011). Among the more important components of atmospheric aerosols is carbon, which on average constitutes 20–30 % of PM10 mass and as much as 50 % of PM2.5 (Kim et al. 2012). Their presence in the atmosphere affects the regional and global water budget, climate change, and agriculture (Satsangi et al. 2012), and there is also substantial evidence of associations between air pollution and human health (Vanos et al. 2015).

Carbonaceous aerosols are durable and may be transported far away from the source. Two carbon forms are usually distinguished: organic carbon (OC) and elemental carbon (EC). EC is emitted directly into the atmosphere as a primary pollutant in the form of fine particles, mainly during incomplete combustion of fossil fuels (traffic, industry, domestic heating, and refuse burning) and biomass burning (Satsangi et al. 2012). OC is a mixture of aliphatic and aromatic compounds, aldehydes, ketones, alcohols, and carboxylic acids, which may derive from both primary and secondary sources (Molnar et al. 1999; Lewandowska et al. 2010). Publications concerning elemental and organic (both primary and secondary) carbon in aerosols of different sizes at rural stations in north-eastern Poland (and even Eastern Europe) are still a rarity. However, variations in the carbon content and form of atmospheric aerosols can be responsible for changes in atmospheric conditions, such as temperature or frequency and volume of precipitation, thus triggering weather anomalies such as droughts or floods (Alexandrov and Hoogenboom 2000). OC in small aerosols also poses a threat to human health (Ren-Jian et al. 2012). Hence, the aim of the present paper was to determine the seasonal and daily variability of elemental and organic carbon concentrations (both primary and secondary) and their contribution to PM2.5 and PM10 under the influence of the changeable atmospheric conditions at Puszcza Borecka (Polish National Nature Reserve). As this rural station is located far away from major emission sources, industrial facilities, urban areas, and busy streets, it is not significantly impacted by them.

The important aspect, which this paper aims to point out, is rather the role of agriculture and vegetation in creating high concentrations of carbon compounds in aerosols. In addition, the paper attempts to indicate the situations in which pollutants are transported from the Polish-Russian border and from cities located up to 50 km away from the study area. Seasonal variability, in terms of the proportions of EC and primary and secondary OC present in PM2.5 and PM10, also demonstrates that the influence of all emission sources—local, regional, and cross-border—can be periodically observed at the rural Diabla Gora station.

## Methods

### Sampling

**Fig. 1:**
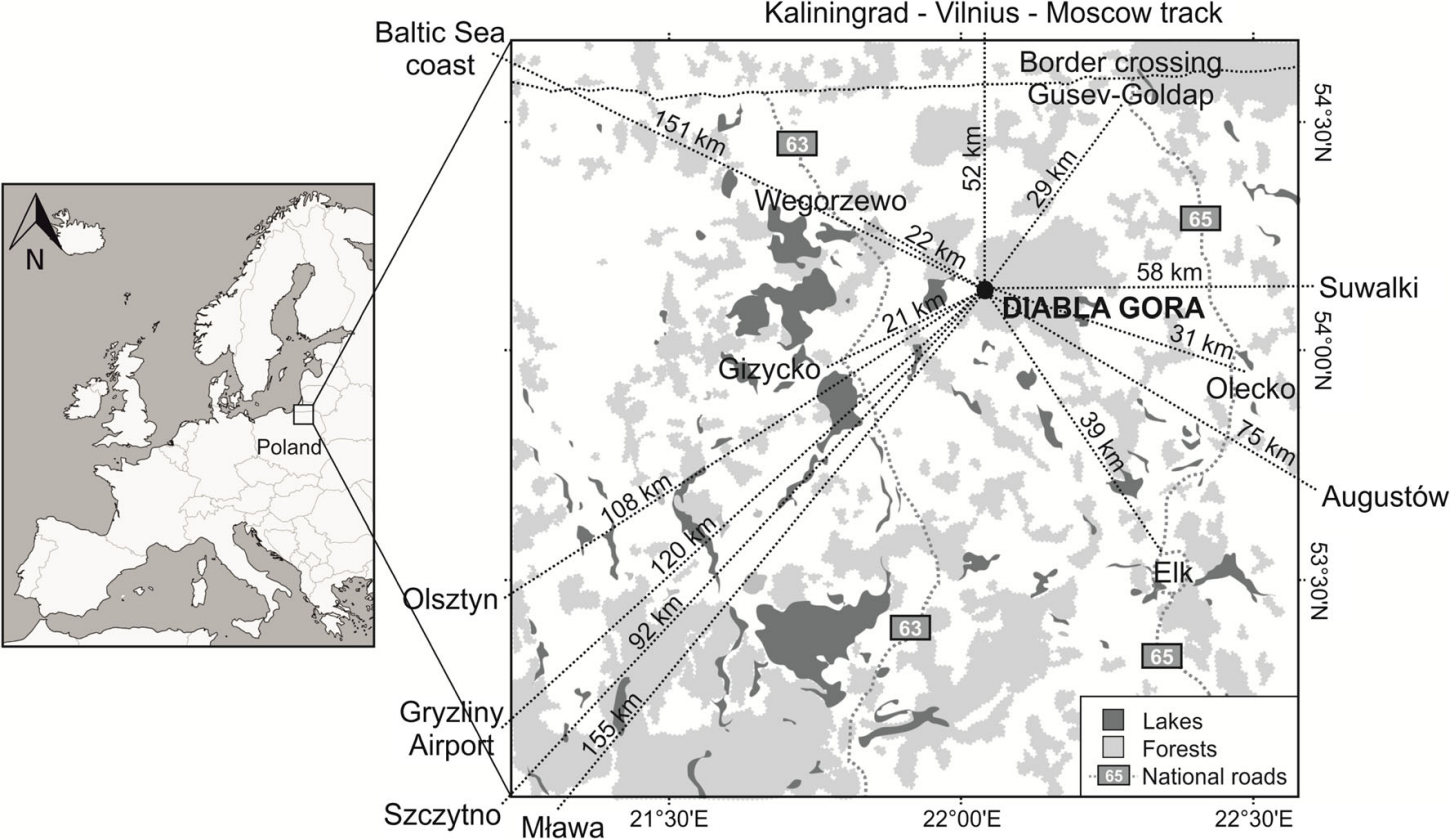
Measurement station in Diabla Gora and surrounding emission sources

Diabla Góra (*φ* = 54° 07′ N, *λ* = 22° 02′ E), a rural station lying on the western edge of the Puszcza Borecka Forest between the Great Masurian Lakes to the west (10–40 km) and the Elk Lake District to the southeast (35 km) (Fig. 1), is one of 3800 stations involved in the World Meteorological Organization’s Global Atmosphere Watch programme and is also part of the Integrated Monitoring Programme and the European Monitoring Evaluation Programme (EMEP). Puszcza Borecka is one of the cleanest places in Poland, located far from urban areas, population centers, industry, and highways, and the land is utilized primarily in agriculture and forestry, with as much as 53 % of its area taken up by forests, 20 % by cultivable land, and 13 % by meadows and pastures. Areas used for human habitation (1.5 %) and transportation (about 3 %) do not constitute a significant proportion of land usage here (Degórska et al. 2006), and the nearest large towns, located at relative distances of 21–22 km to the west of the measuring station, are Gizycko and Wegorzewo (Fig. 1), while Suwalki, Olecko, and Elk are situated to the east and southeast (58, 31, and 39 km away, respectively) and several other large towns (Olsztyn, Szczytno, and Mlawa) are located further away (92–155 km) to the southwest. There is also a border crossing with Russia (Goldap) located to the northeast. There are few transport routes in the immediate vicinity of the measuring station although road no. 63, which connects two border crossings with major roads in northeastern Poland, runs approximately 20 km west of Diabla Gora and road no. 65, running directly northwards to the Kaliningrad Oblast border crossing at Goldap, is situated to the east. The station is located 151 km from the Baltic Sea coast (Fig. 1).

Samples of PM2.5 and PM10 were collected in 24-h cycles between 1 January and 31 December 2009. In total, 354 samples of PM2.5 and 365 of PM10 were collected, with OC and EC being determined in each. The concentration of basic ions (K^+^, Na^+^, NH_4_^+^, Ca^2+^, Mg^2+^, Cl^−^, NO_3_^−^, SO_4_^2−^) was also measured in order to determine the origins of the different carbon forms in aerosols of both sizes.

Collection of PM2.5 and PM10 was performed using a high-volume DIGITEL DHA-80 aerosol sampler with air flow at 30 m^3^ h^−1^. The sampler was in accordance with the EN 12341 norm and the VDI/DIN 2463 directive on collection of PM10, as testified to by a certificate issued by the UMEG in Germany. Quartz filters of 150 mm diameter were used, heated at 550 °C using a furnace in order to eliminate volatile impurities and reduce blank values. Before and after use, the filters were conditioned at a constant temperature in a desiccator and then weighed with an accuracy of 10^−5^ g at a temperature of 20 ± 1 °C and air humidity of 50 ± 5 %. Thereafter, aerosol mass was determined for each filter by calculating the difference between the obtained weights (μg) and then dividing by the volume of filtered air. The final concentration results for PM2.5 and PM10 were thus given in micrograms per cubic meter of air.

### Meteorological information

Meteorological data was collected throughout the measurement period with the use of an automatic Milos 500 station by Vaisala. The average values and standard deviations (SD) obtained for the meteorological parameters (annual, monthly, heating period, non-heating period) are presented in Table 1. The combined measurement results for wind speed, air temperature, and air humidity were averaged based on 1-h values, while precipitation is presented as a monthly sum.

**Table 1:** Statistical characteristic of the meteorological conditions in Diabla Gora during the measurement period (1 January–31 December 2009)

Period	T^a^ ± SD^b^	Rh^c^ ± SD^b^	Vw^d^ ± SD^b^	*Σ* H^e^
January	−3.6 ± 4.7	92.4 ± 7.8	1.8 ± 1.4	29.4
February	−2.6 ± 5.6	93.2 ± 4.5	1.9 ± 1.4	41.3
March	0.8 ± 2.3	87.0 ± 8.5	2.4 ± 1.4	65.4
April	9.1 ± 4.0	59.6 ± 15.8	2.3 ± 0.9	3.9
May	11.9 ± 2.9	72.8 ± 9.9	2.9 ± 1.1	66.3
June	14.4 ± 4.0	83.7 ± 8.1	2.3 ± 0.7	159.7
July	16.2 ± 4.4	80.3 ± 7.9	2.2 ± 0.7	75.1
August	16.5 ± 2.4	76.9 ± 9.3	2.0 ± 0.8	55.3
September	13.9 ± 2.8	79.6 ± 7.1	2.3 ± 1.2	23.3
October	5.3 ± 3.1	91.1 ± 6.5	2.4 ± 1.4	80.8
November	4.0 ± 2.6	91.7 ± 8.0	3.1 ± 1.5	53.9
December	−2.7 ± 6.3	91.7 ± 6.1	2.3 ± 1.5	40.8
Non-heating^f^	13.3 ± 4.6	75.8 ± 12.5	2.4 ± 1.5	402.7
Heating^g^	0.1 ± 5.3	91.5 ± 7.0	2.3 ± 0.9	292.5
Annual mean ± SD^b^	6.9 ± 8.3	83.3 ± 12.9	2.3 ± 1.2	4.1
Annual min.^h^	−16.9	37.1	0.3	0.1
Annual max.^i^	25.1	100.0	6.8	37.5

^a^Temperature [°C]

^b^Standard deviation

^c^Relative humidity [%]

^d^Wind speed [m s^−1^]

^e^Sum of precipitation [mm]

^f^Period between April and September 2009

^g^Period between January and March 2009 and between October and December 2009

^h^Minimal concentration

^i^Maximal concentration

**Fig. 2:**
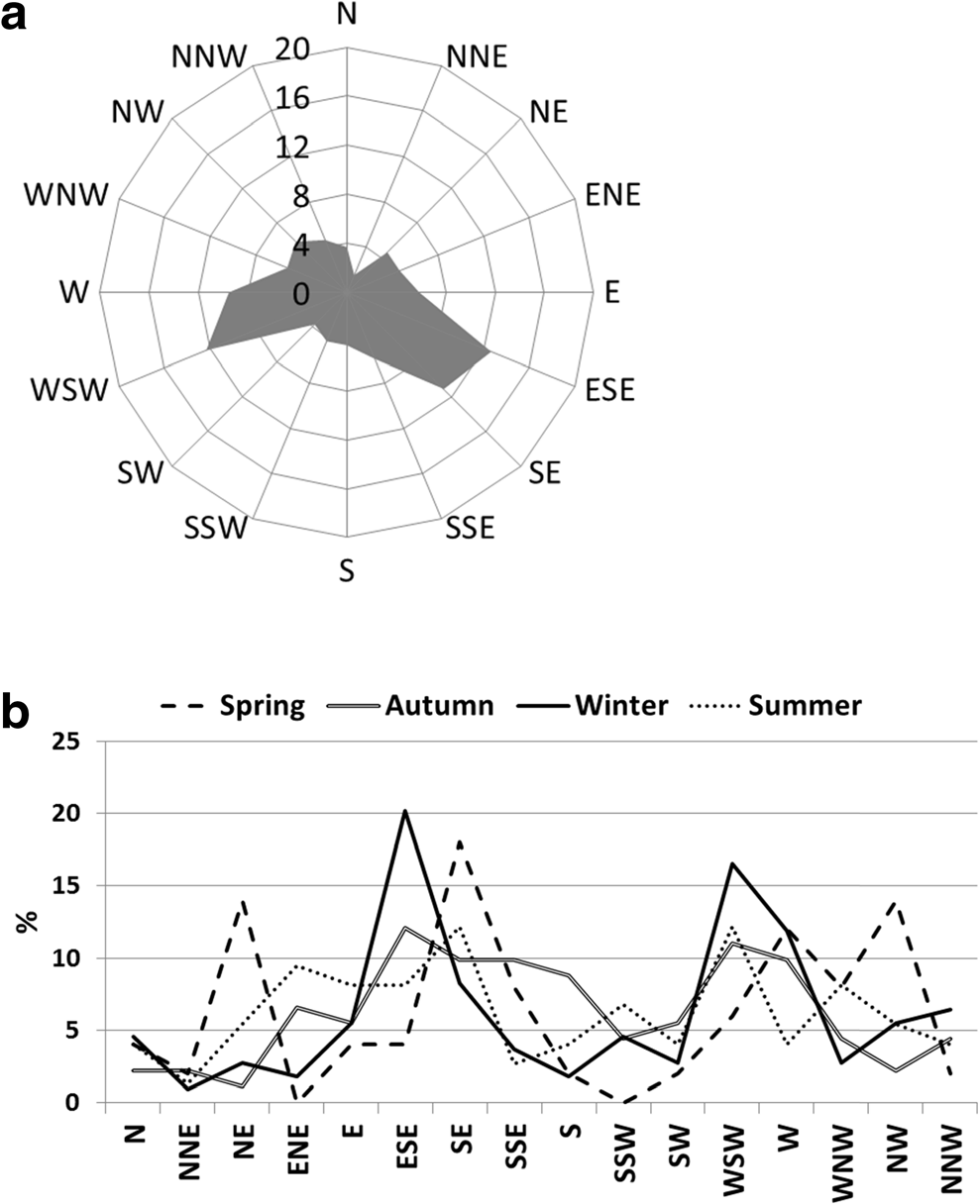
The predominant directions of advection [%] determined on the basis of the air mass trajectories **a** in 2009 and **b** in the particular seasons of 2009 at the Diabla Gora station

For each measurement period, air mass backward trajectories were determined using the Global Data Assimilation System (GDAS) meteorological dataset and model-calculated vertical velocities HYSPLIT model. This is a complete system for computing air parcel trajectories to complex dispersion and deposition simulations (Draxler and Hess 1997). Forty-eight-hour air mass backward trajectories were determined at 6 a.m. UTC (measurement start time) at 6-h intervals, at heights of 500, 1000, and 1500 m for a given period. If subsequent trajectories were found to be significantly different, an additional trajectory was determined between them over a 3-h period. These additional trajectories were set at the collection height. Each trajectory was compared with the predominant wind direction obtained by the Vaisala station, and, on the basis of both, the predominant directions of advection were established for the entire year of 2009 and the individual seasons (Fig. 2). The volume of precipitation in air masses en route from their source, as well as the height of the mixing layer, was also assessed.

### Chemical analysis of carbon species (OC, EC) and ions in PM2.5 and PM10

OC and EC in PM2.5 and PM10 aerosols collected in Diabla Gora were analyzed using a Sunset Laboratory Dual-Optical Carbonaceous Analyzer which meets the requirements of NIOSH 5040, IMPROVE, and EUSSAR2 protocols. Studies conducted by Cavalli et al. (2010) have demonstrated that differences in temperature range and residence times at each temperature step might significantly alter the measured amounts of OC and EC using different protocols. Internal calibration showed significant differences in OC and EC values when analyzing these same samples (*n* = 30) using the IMPROVE and EUSAAR2 methods. Results obtained by the NIOSH method for OC were as much as 32.7 % lower and for EC up to 27.2 % higher than using the EUSAAR2 method. In the present study, the EUSAAR2 protocol was applied to the analysis owing to the optimal maximum temperature (650 °C) obtained at the end of the first stage, which caused just 2.5 ± 2.4 % of EC to be combusted during the first stage of analysis (Cavalli et al. 2010).

A rectangular piece of a quartz filter with a surface area of 1.5 cm^2^ was placed in a quartz oven, where it was dually analyzed. The method’s limit of detection for 72 blanks was in the order of 0.3 μg per 1 cm^2^ of the filter (a level of 0.02 μg m^−3^) for both OC and EC, while the analytical error was <6 % for EC and <10 % for OC (with a 99 % confidence interval). All carbon analysis results for the environmental samples were reduced by the values for blank samples and took into account air flow through the high-volume sampler (HVS) (30 m^3^ h^−1^). The blank sample value for OC did not exceed 3.0 μg per 1 cm^2^ of the filter (0.2 μg m^−3^), while for EC it was below the detection limit of the method. Apart from automatic calibration (internal standard—5.0 % methane in equilibrium with analytically pure He), which took place at the end of the second stage of analysis, an external standard (99 % analytically pure sugar solution) was analyzed after every 10–15 samples. The analytical error of external calibration was on average 4.5 %. Inter-laboratory comparison was also performed (with Université du Québec a Montréal) using the isotopic ^13^C method on a 2500 NC Elemental Analyzer. The agreement between the two methods was confirmed by a high Pearson correlation coefficient value for total carbon (*r* > 0.9).

To determine the sources of origin for carbon species found in PM2.5 and PM10 over Diabla Gora, some concentrations of ions were needed. The analysis of ionic aerosol components corresponding to the soluble fraction was carried out using the ionic chromatography method on a Metrohm 850 Professional IC chromatograph, equipped with an Asupp7 column for anions (250 mm) and a C4 column (150 mm) for cations, both with protective guard columns. The chromatograph was also equipped with a conductometric detector and a chemical three-bed Micro Packed Bed Suppressor. In the anion analysis method, 3.6 M Na_2_CO_3_ was used as an eluent, while for cations the eluent was a mixture of the following acids: 1.7 mmol dm^−3^ of HNO_3_ and 0.7 mmol dm^−3^ of dipicolinic acid (DPA). The Milli-Q water used for the preparation of eluents had electrical conductivity <0.1 μS cm^−1^. To correct the concentrations of ionic species, field blanks were used. The detection limits were as follows: 0.24 μg m^−3^ for Cl^−^, 0.70 μg m^−3^ for NO_3_^−^, Na^+^, Ca^2+^, NH_4_^+^, Mg^2+^, and K^+^, and 1.00 μg m^−3^ for SO_4_^2−^. Each analysis took into account a minimum of 20 blanks and the medium air flow through the HVS sampler (30 m^3^ h^−1^). Replicate checks were performed after every 10–15 samples. Relative standard deviations (RSD) of replicate analyses were calculated at 0.5–2.0 % for Cl^−^, 0.6–2.5 % for NO_3_^−^, 0.1–2.3 % for SO_4_^2−^ and PO_4_^3−^, 0.7–3.4 % for Na^+^ and Ca^2+^, 1.4–2.0 % for NH_4_^+^, and 2.3–3.5 % for K^+^.

After sampling, the quartz filter (150 mm in diameter) was always weighed and then two pieces each 12 mm in diameter were cut out, one for the analysis of cations and the other for anions. Having been prepared in such a way, the samples were then placed in polyethylene tubes (cations and anions separately) with 12 cm^3^ of Milli-Q water and inserted into an ultrasonic bath (Sonic 10) at 37 °C for 30 min in order to extract the ions into the solution. To analyze cations, 12 μl of 2 M of nitric acid was added per 12 cm^3^ of each sample prior to insertion into the ultrasonic bath.

Having been removed from the ultrasonic bath, the samples were then drained through a membrane filter with 0.45 μm pore diameter and placed in an 858 Professional IC sample processor, which is equipped with an on-line filtration process, ensuring additional filtration of samples through a 0.20-μm filter. Cations and anions were analyzed separately on IC.

### Other calculations

The concentration of sea salt aerosols [μg m^−3^] was calculated assuming the value of 1.47 to represent the proportion of K^+^, Mg^2+^, Ca^2+^, SO_4_^2−^, and HCO_3_^−^ ions to sodium in the sea water (Quinn et al. 2002):

1 $$ Ass=C{l}^{-}+\left(1.47\bullet N{a}^{+}\right) $$

The actual concentration of non-sea salt sulfates, non-sea salt potassium, and non-sea salt calcium ions in aerosols [μg m^−3^] was calculated according to the following relations (Wang et al. 2005; Sen-chao et al. 2007):

2 $$ nssS{O}_4^{2-}=S{O}_4^{2-}-\left(0.06\bullet N{a}^{+}\right) $$

3 $$ nssC{a}^{2+}=C{a}^{2+}-\left(0.0373\bullet N{a}^{+}\right) $$

4 $$ nss{K}^{+}={K}^{+}-\left(0.0355\bullet N{a}^{+}\right) $$

The molar ratio of NO_3_^−^/SO_4_^2−^ was taken to be an indicator of the relative importance of stationary to mobile sources of sulfur and nitrogen in the atmosphere (Sen-chao et al. 2007).

## Results and discussion

### Annual organic and elemental carbon concentration in PM2.5 and PM10

In the atmosphere over Diabla Gora (Puszcza Borecka), the mean annual concentrations of PM2.5 and PM10 in 2009 were equal to 13.4 ± 11.6 and 18.2 ± 12.7 μg m^−3^, respectively (Table 2). The obtained results were similar to those observed at other rural stations in Europe including those gathered by Putaud et al. (2010), who conducted PM2.5 and PM10 aerosol measurements at over 60 stations, spread across Europe and of varying character, for over 10 years. At the rural stations, the average PM2.5 concentration ranged between 10 and 24 μg m^−3^, while for PM10 it was between 15 and 39 μg m^−3^. Slightly higher PM2.5 concentrations (15 μg m^−3^) were noted in 2010 by Rogula-Kozłowska et al. (2014) at the same Diabla Gora station, as a result of a much colder winter. That year, the temperature in Poland periodically dropped to −30 °C and was two times lower than in 2009 (Table 1), resulting in a higher average PM2.5 concentration in winter 2010 than in winter 2009 (25.9 μg m^−3^ compared to 19.3 μg m^-3^, in 2009). In the non-heating season, values were almost the same in both years (10.7 and 10.6 μg m^−3^ in 2009 and 2010, respectively).

**Table 2:** Statistical characteristic of PM2.5 and PM10 mass concentration and organic and elemental carbon [μg m^−3^] in Diabla Gora in 2009

Season		PM2.5			PM10		
		Mean^a^ ± SD^b^	Min.^c^	Max.^d^	Mean^a^ ± SD^b^	Min.^c^	Max.^d^
PM	Spring	8.3 ± 6.8	0.3	32.2	11.7 ± 7.2	2.9	35.5
	Summer	10.2 ± 4.5	1.4	28.9	15.0 ± 7.8	3.7	52.6
	Autumn	14.4 ± 10.7	3.7	69.0	21.3 ± 14.6	5.6	75.9
	Winter	19.3 ± 15.6	2.2	99.4	24.1 ± 15.0	6.7	104.8
	Non-heating^f^	10.7 ± 7.9	0.4	69.0	16.0 ± 11.1	3.3	75.9
	Heating^g^	16.3 ± 14.1	0.3	99.4	20.5 ± 14.0	2.9	104.8
	Annual	13.4 ± 11.6	0.3	99.4	18.2 ± 12.7	2.9	104.8
OC	Spring	2.8 ± 2.1	0.2	10.8	3.6 ± 2.3	0.5	12.5
	Summer	1.8 ± 1.0	0.2	4.4	2.7 ± 1.2	0.9	6.0
	Autumn	4.3 ± 3.9	0.6	27.7	4.7 ± 4.5	<LD^e^	30.3
	Winter	5.9 ± 6.2	0.5	35.3	6.4 ± 5.1	0.6	44.4
	Non-heating^f^	2.7 ± 2.6	0.2	27.7	3.4 ± 3.1	<LD^e^	30.3
	Heating^g^	5.2 ± 5.0	0.4	35.3	5.2 ± 4.5	0.5	44.4
	Annual	3.9 ± 3.7	0.2	35.3	4.3 ± 3.9	<LD^e^	44.4
EC	Spring	0.6 ± 0.4	0.1	1.4	0.6 ± 0.3	<LD^e^	1.6
	Summer	0.2 ± 0.1	<LD^e^	0.6	0.3 ± 0.1	<LD^e^	0.6
	Autumn	0.9 ± 0.8	0.1	5.6	1.0 ± 0.9	0.1	5.7
	Winter	0.9 ± 0.6	0.2	3.8	1.0 ± 0.6	0.2	4.1
	Non-heating^f^	0.5 ± 0.4	0.1	5.6	0.5 ± 0.4	<LD^e^	5.7
	Heating^g^	0.8 ± 0.6	0.1	3.8	0.9 ± 0.5	<LD^e^	4.1
	Annual	0.7 ± 0.6	<LD^e^	5.6	0.7 ± 0.6	<LD^e^	5.7

^a^Average concentration

^b^Standard deviation

^c^Minimal concentration

^d^Maximal concentration

^e^Concentration below the limit of detection

^f^Period between April and September 2009

^g^Period between January and March 2009 and between October and December 2009

In the atmosphere over the rural station in Diabla Gora, the shared origin of PM2.5 and PM10 has been confirmed by statistically relevant Spearman correlation coefficients (PM2.5/PM10, *r* = 0.83 Spearman, *p* < 0.05), with carbon compounds found to be major contributors to the mass of both species (34.5 and 27.5 %, respectively). Rogula-Kozłowska et al. (2014) reported a similar share of carbon compounds in PM2.5 (33 %) over the same station in 2010. Results obtained in the urbanized coastal region of the Baltic Sea (Gdynia station) in 2008–2009, however, demonstrated a higher contribution of carbon compounds in PM10 (42 %) (Lewandowska and Falkowska 2013). In general, the contribution of total carbon to aerosol mass has proven to be as much as 20 % lower at rural stations than at urban or industrial stations (Sánchez de la Campa et al. 2009; Rogula-Kozłowska et al. 2014; Zhu et al. 2014). In the coastal zone of the Baltic Sea, the higher contribution of carbon in PM10 may be combined with biological activity in the sea (Lewandowska and Falkowska 2013).

The concentration of EC in both aerosol fractions over the analyzed station was the same, amounting on average to 0.7 ± 0.6 μg m^−3^, while OC exhibited an insignificantly higher mean concentration in PM10 than in PM2.5 (4.3 ± 3.9 and 3.9 ± 3.7 μg m^−3^, respectively) (Table 2). The concentrations of both carbon fractions in PM2.5 and PM10 were comparable to those obtained at other rural stations in Europe. For example, in 2005–2006, in the atmosphere over the southern part of Spain, concentrations of OC and EC in PM2.5 amounted to 2.8 and 0.6 μg m^−3^, respectively, and in PM10 they were equal to 3.0 and 0.6 μg m^−3^, respectively (Sánchez de la Campa et al. 2009). Meanwhile, at a rural station in Central England in the same year, the concentration of OC ranged between 3.2 and 3.6 μg m^−3^ and for EC between 0.9 and 1.0 μg m^−3^, for PM2.5 and PM10, respectively (Harrison and Yin 2008). At a rural station in Morogoro (Tanzania) in 2011, Mkoma et al. (2013) noted concentrations of OC (3.7 μg m^−3^) and EC (0.8 μg m^−3^) in PM2.5 similar to those at Diabla Gora while concentrations in PM10 were slightly higher (6.8 μg m^−3^ for OC and 0.6 μg m^−3^ for EC). Both carbon fraction concentrations obtained in Diabla Gora were lower in comparison to the Raciborz rural station, which is located in the more polluted southern part of Poland. During 5 months of PM1 measurements (August–December 2010), the authors of that study noted EC concentrations between 0.14 and 5.13 μg m^−3^ and OC concentrations between 2.7 μg and 25.8 μg m^−3^ (Rogula-Kozłowska and Klejnowski 2013).

### Primary and secondary organic carbon concentrations in PM2.5 and PM10

**Fig. 3:**
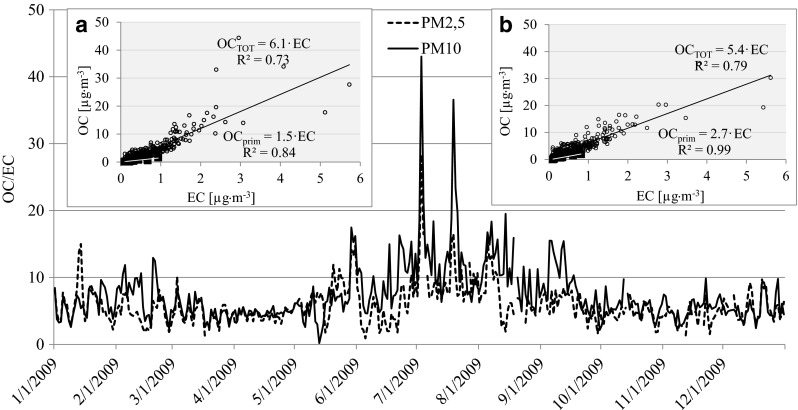
The variability of the OC/EC ratio in PM2.5 and PM10 aerosols in 2009 at the Diabla Gora station and the dependency between the concentration of total and primary OC [μg m^−3^] with EC [μg m^−3^] in PM2.5 (**a**) and in PM10 (**b**)

In the atmosphere over Diabla Gora, the mean percentage of EC in PM2.5 amounted to 5.2 % and in PM10 to 3.7 %. The OC exhibited higher proportions, constituting respective levels of 29.3 % in PM2.5 and 23.8 % in PM10. Such a percentage is typical for both fractions in Europe, where EC comes mainly from primary sources such as incomplete combustion emissions from vehicle exhaust (diesel or gasoline-based engines), biomass burning and coal combustion (Pio et al. 2008). Therefore, OC emissions can most probably be ascribed to the above cited primary sources and, in addition, to secondary processes occurring in the atmosphere such as the many complex oxidation processes of volatile organic compounds including natural sources (VOC) (Guor-Chen et al. 2008). The measurements made as part of the present study indicated that OC and EC originated from the same source in both aerosol fractions (OC/EC *r* = 0.87, *p* < 0.05 for PM2.5 and OC/EC *r* = 0.81, *p* < 0.05 for PM10). However, in order to determine the origin of primary and secondary organic carbons in aerosols, the value of the OC/EC ratio was calculated for each individual sample (Na et al. 2004; Harrison and Yin 2008). The secondary organic carbon fraction in aerosols (SOC) was calculated by determining the lowest trend line on the plot of the relationship between OC and EC, thereby obtaining an equation describing this relationship (Fig. 3). All points located below the line represent the concentrations of primary organic carbon (OC_prim_), testifying towards primary sources of origin, as in the case of EC. Meanwhile, the points above the line of the lowest OC/EC ratio values signify SOC, the concentration of which was determined based on the difference between total OC and OC_prim_ (Harrison and Yin 2008). The obtained equation OC_prim_ = 1.5 EC for PM2.5 (Fig. 3) suggests an almost constant relationship between OC_prim_ and EC and a common source of origin in smaller particles. Interestingly, an identical relationship was observed by Lewandowska et al. (2010) at a station in Gdynia, located in an urbanized coastal area of the sea, in PM10, and by Lin and Tai (2001) in the city of Kaohsiung, located on the South China Sea in Taiwan for PM2.5. In PM10 in Diabla Gora, an additional source of primary OC, more clearly marked than in Gdynia or in Taiwan, was noticed since the equation took the form of OC_prim_ = 2.7 EC. However, in both aerosol fractions in the atmosphere over Puszcza Borecka, SOC prevailed in the total OC fraction (74.1 % OC in PM2.5 and 60.1 % OC in PM10).

**Fig. 4:**
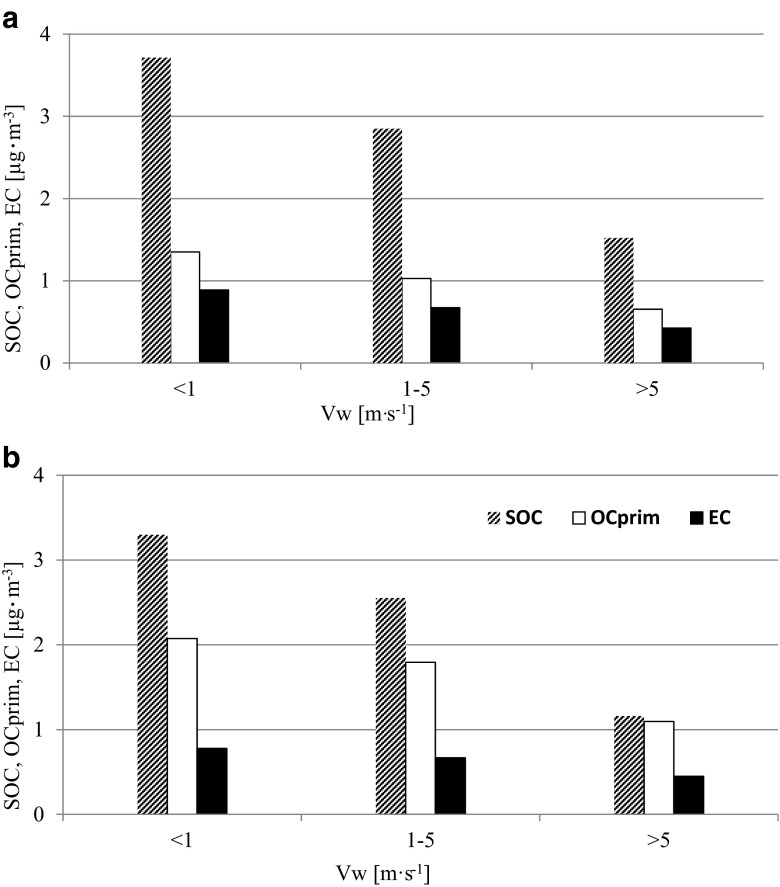
The variability of the concentrations of secondary organic carbon (SOC), primary organic carbon (OC_prim_), and elemental carbon (EC) [μg m^−3^] in PM2.5 (**a**) and in PM10 (**b**) in Diabla Gora in 2009 at various wind speeds (Vw) [m s^−1^]

Additionally, it was found that, regardless of the carbon species and aerosol diameter, the concentrations increased whenever wind speed dropped (Fig. 4). Smaller SOC, OC_prim_, and EC concentrations were observed in PM2.5 and PM10 with the highest wind speed (>5 m s^−1^), while the highest values were obtained at wind speed of no more than 1 m s^−1^. This was linked to local emission sources and low dispersion of pollutants. High concentrations, particularly of primary and SOC, were also observed when air masses drifted over the station, carried by wind at between 1 and 5 m s^−1^ of regional character (Lim and Turpin 2002; Lewandowska and Falkowska 2013). Only OC_prim_ concentrations were higher in PM10 than in PM2.5 (Fig. 4). Detailed analysis of the concentrations and source regions for all carbon species indicated that the reason for this was local agriculture and vegetation activity in surrounding farmland and forests (Fig. 1).

In terms of the other carbon species, the increase in concentration was more defined in PM2.5 than in PM10, and this resulted from polluted air masses being carried from over nearby habitations at medium wind speed (Fig. 4). The nearest of these is the town of Gizycko, located 21 km to the west-southwest (WSW) of the station, while four other towns lie between 20 and 80 km away to the east-southeast (ESE) and south-southeast (SSE): Elk, Augustow, Olecko, and Suwalki. Of these, Olecko and Elk are the closest (respectively 31 and 39 km, Table 1, Fig. 1). Gizycko and Wegorzewo (21 and 22 km from the station, respectively) are both crossed by trunk road no. 63, which connects two border crossings with major road corridors in northeastern Poland and has a traffic level of 13,000 vehicles per 24 h. Both Elk and Olecko lie on the route of trunk road no. 65, which leads to the Kaliningrad Oblast border crossing at Goldap (Fig. 1). The traffic on that road can reach 21,000 vehicles per 24 h. Additionally, although the other towns are more tourism-oriented (holiday resorts, aeroclub, water sports), Elk is of an industrial nature and features factories which produce doors, paper, and boards, as well as an established wood industry.

### Seasonal variability of the concentrations of primary and secondary organic carbon in PM2.5 and PM10

**Fig. 5:**
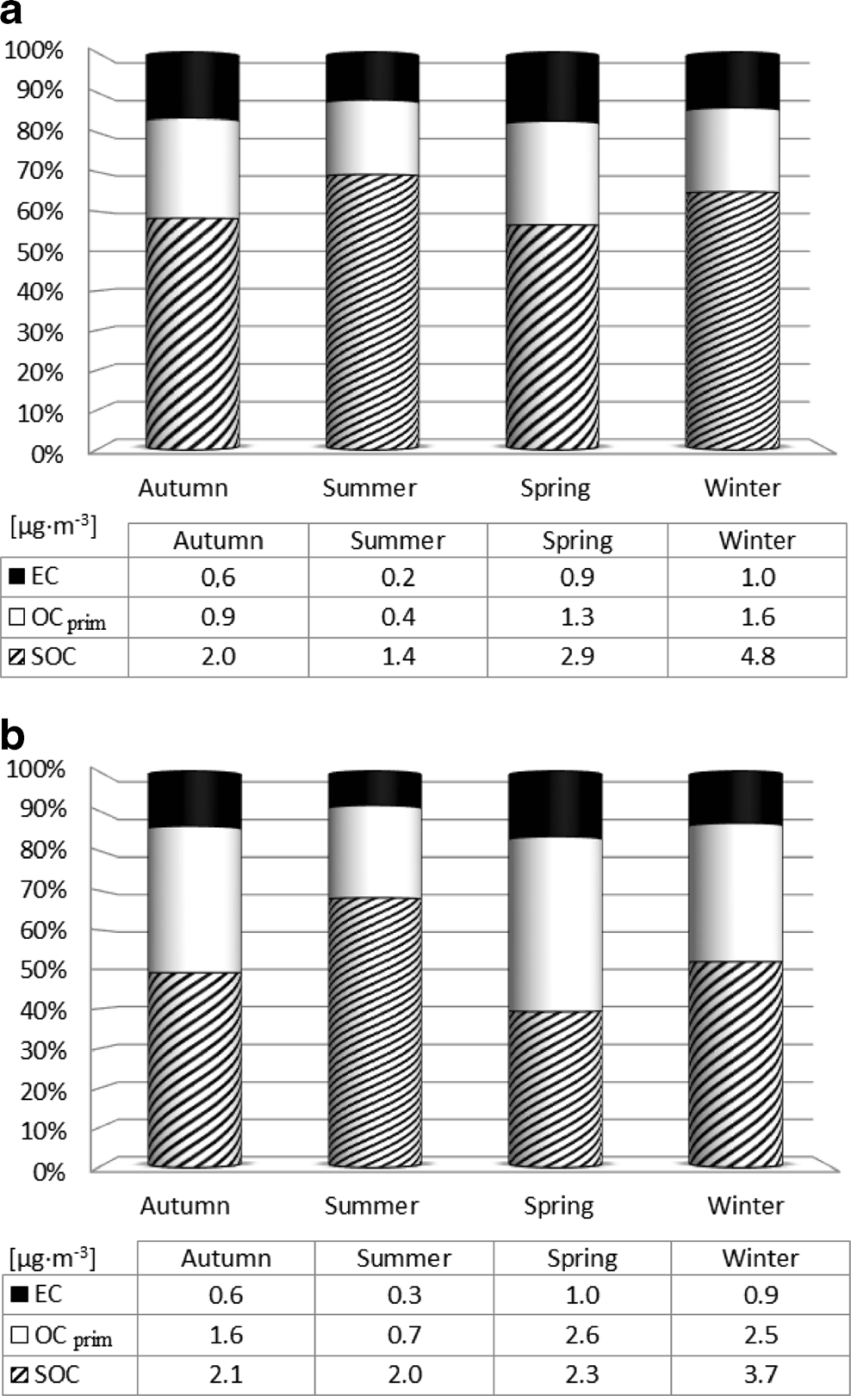
Seasonal changes in the concentration [μg m^−3^] and proportions [%] of primary (OC_prim_) and secondary organic carbon (SOC) and elemental carbon (EC) in the total carbon fraction of PM2.5 (**a**) and PM10 (**b**) in Diabla Gora in 2009

The influence of the particular emission sources on elemental, primary, and secondary carbon concentrations in PM2.5 and PM10 at the rural Diabla Góra station in 2009 was clearly pronounced when seasonal divisions were considered (Fig. 5). The individual seasons were selected according to the calendar and adjusted for prevailing meteorological conditions (spring 26 March–31 May 2009; summer 1 June–31 August 2009; autumn 1 September–30 November 2009; winter 1 January–25 March 2009 and 1 December–31 December 2009). The concentrations of all carbon fractions in PM2.5 decreased in the following order: winter > spring > autumn > summer. In PM10, such a tendency was observed only for SOC, while OC_prim_ and EC concentrations decreased as follows: spring > winter > autumn > summer (Fig. 5). With the exception of spring 2009, though, SOC always prevailed over OC_prim_.

It was found that in the winter months of 2009, the aerosols measured in Diabla Gora had a shared source of organic and elemental carbon (*r* = 0.88, *r* = 0.81, PM2.5 and PM10, respectively). The highest concentrations of SOC in both PM2.5 and PM10 occurred during that period, reaching 13.4 and 7.6 μg m^−3^, respectively. SOC is very stable in winter, as it does not evaporate from aerosols as easily as during the warm period of the year (Jones and Harrison 2005). In Diabla Gora, the highest concentrations of SOC in winter were observed when local and regional air masses were transported with advection from ESE and WSW (Fig. 2) with the wind speed no higher than 2.5 m s^−1^. At low air temperatures and thermal inversion, the vertical movement of air was limited, causing pollutants to accumulate close to the ground and not to become dispersed over very long distances. In the described cases, pollutants were transported over Diabla Gora from nearby towns (Olecko, Gizycko, and Elk), located no further than 40 km away from the measurement station (Fig. 1). High SOC concentration in winter was the consequence of coal combustion in the communal sector, and this appears to be the most important source of carbon in the air over Poland (NEM 2009). An increase in SOC concentration was also noted at the same advections in early spring, when the heating season was still ongoing (8.3 μg m^−3^ in PM2.5 and 6.0 μg m^−3^ in PM10), and one can therefore reach the conclusion that in the air over the station during the heating season, there is an increase in the proportion of “ageing” secondary aerosols, formed as a result of the transformations of primary OC of regional origin.

**Fig. 6:**
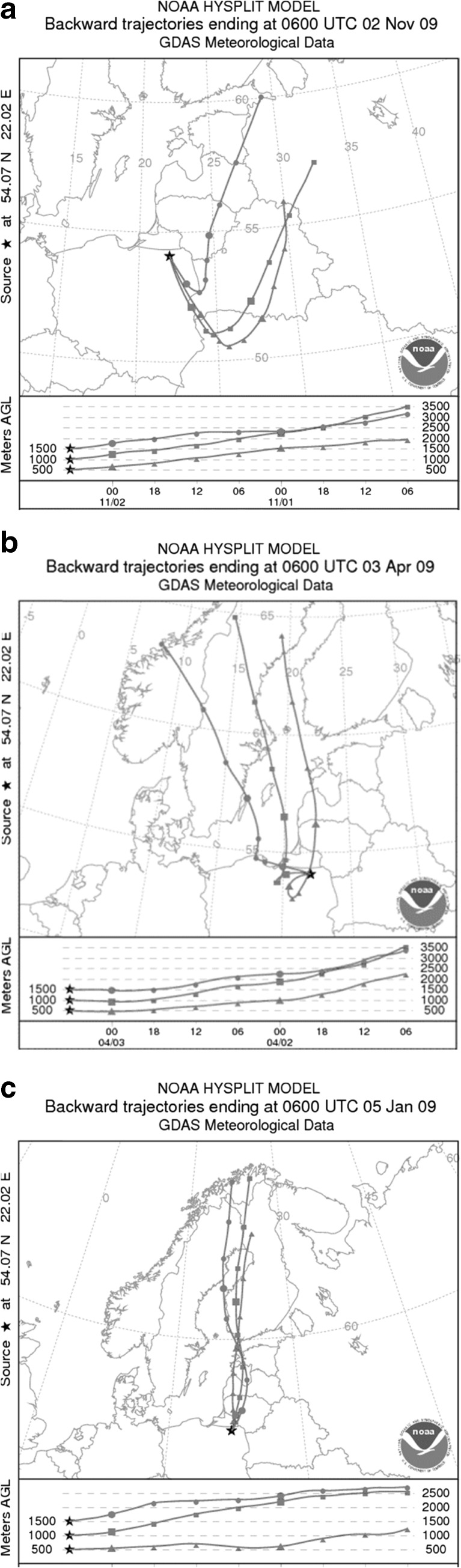
Chosen examples of inflow of air masses from over cemeteries (**a**), from Elk city (**b**), and from the Polish-Russian border at Goldap (**c**)

During the winter months, an increase in EC concentration was also noticed, occurring at the same advection as for SOC. In this case, the EC value was found to be almost twice the annual mean (1.3 and 1.2 μg m^−3^ in PM2.5 and PM10, respectively) and could be a consequence of traffic in the nearest villages and towns (21–31 km). An interesting case involving an increase in EC concentration (1.0 and 1.1 μg m^−3^ in PM2.5 and PM10, respectively) was also observed on 2 November 2009, following All Saints Day. The Polish tradition is to visit cemeteries and burn large numbers of votive candles on 1 and 2 November, and thus the high concentration of pollutants was a result of the inflow of air masses from over cemeteries located to the SE of the station (Figs. 1 and 6a). At that time, the NO_3_^−^/SO_4_^2−^ ratio was also high, showing values of 4 for PM2.5 and 4.5 for PM10. We can reasonably assume that the dominant source of EC in this instance was the traffic travelling to and from nearby cemeteries. However, the average NO_3_^−^/SO_4_^2−^ ratio was lower than 1.0 (in average 0.5–0.6 depending on fraction) indicating stationary sources to be more important in Diabla Gora than transportation (Sen-chao et al. 2007).

Spring turned out to be the most interesting period in terms of diversity of sources of carbon in aerosols. The primary carbon fraction was found to be predominant in PM10, but the increase in concentration of OC_prim_ was also visible in PM2.5 (Fig. 5). In Poland and other central-eastern European countries, spring is generally a period of intense vegetation but also agricultural activity (including fertilization of the fields) and biomass burning (e.g., grass burning). It is unsurprising then that the greatest influence in Diabla Gora during spring came from the transport of air masses from forested and agricultural areas (Figs. 1 and 2a), and a rise observed in potassium in aerosols at that time (Table 3) could be due to the enrichment of soil with this element and its subsequent lifting by the wind into the atmosphere. On the other hand, potassium could also be associated with biomass burning from agricultural activities. In the springtime, ammonium nitrate, a key component of mineral fertilizers, was also determined in aerosols of both fractions (NH_4_^+^/NO_3_^−^ *r* > 0.80 for PM2.5 and PM10). Ammonium ions and nitrates together constituted 15.7 % of PM2.5 mass and 11.2 % of PM10 mass. A similar situation was observed during autumn when air masses were transported from over agricultural areas (Figs. 1 and 2). During spring, as a further consequence of agricultural activity, the highest value of Ca^2+^ concentration in PM10 was also noted. Ca^2+^ was principally in the form of nssCa^2+^ (91.3 % in PM2.5 and 96.7 % in PM10; Table 3), and this may be attributed to the fact that in Poland, owing to the high acidity of soil, lime (calcium carbonate) is used after the winter ploughing. This would explain why the concentrations of nssCa^2+^ were observed to have decreased from spring to autumn (Table 3).

**Table 3:** Indicators of organic and elemental carbon source origin in PM2.5 and PM10 in Diabla Gora in 2009

Period	PM2.5								
	Ass^a^	nssSO_4_^2−b^	nssCa^2+c^	nssK^+d^	nssCa^2+^/OC	nssCa^2+^/EC	nssK^+^/OC	nssK^+^/EC	NO_3_^−^/SO_4_^2−^
Spring	0.62 ± 0.19	1.47 ± 0.97	0.17 ± 0.10	0.17 ± 0.18	0.06	0.38	0.05	0.21	0.63
Summer	0.97 ± 0.46	0.97 ± 0.54	0.11 ± 0.11	0.09 ± 0.08	0.11	0.54	0.07	0.40	0.19
Autumn	0.151 ± 0.49	1.25 ± 1.09	0.04 ± 0.04	0.02 ± 0.02	0.03	0.11	0.01	0.03	0.62
Winter	1.27 ± 1.01	3.24 ± 2.90	0.11 ± 0.10	0.16 ± 0.18	0.05	0.15	0.03	0.15	0.34
Non-heating^e^	0.92 ± 0.43	1.12 ± 0.77	0.12 ± 0.12	0.13 ± 0.12	0.08	0.41	0.05	0.24	0.36
Heating^f^	1.38 ± 0.90	2.63 ± 2.59	0.09 ± 0.09	0.14 ± 0.13	0.04	0.13	0.03	0.17	0.52
Annual	1.14 ± 0.73	1.84 ± 1.72	0.11 ± 0.11	0.14 ± 0.12	0.06	0.29	0.04	0.21	0.50
Period	PM10								
	Ass	nssSO_4_^2−^	nssCa^2+^	nssK^+^	nssCa^2+^/OC	nssCa^2+^/EC	nssK^+^/OC	nssK^+^/EC	NO_3_^−^/SO_4_^2−^
Spring	0.77 ± 0.27	3.01 ± 2.41	0.52 ± 0.52	0.46 ± 0.45	0.20	0.98	0.05	0.58	0.64
Summer	1.19 ± 0.71	1.89 ± 1.32	0.44 ± 0.37	0.25 ± 0.24	0.18	1.63	0.11	0.86	0.33
Autumn	1.54 ± 0.68	1.60 ± 1.29	0.14 ± 0.19	0.09 ± 0.05	0.05	0.35	0.04	0.18	0.76
Winter	1.46 ± 1.04	6.18 ± 4.52	0.25 ± 0.26	0.17 ± 0.16	0.14	0.60	0.04	0.19	0.80
Non-heating^e^	1.11 ± 0.63	2.15 ± 1.83	0.42 ± 0.41	0.32 ± 0.31	0.17	1.22	0.08	0.57	0.48
Heating^f^	1.48 ± 0.94	4.68 ± 4.29	0.22 ± 0.13	0.15 ± 0.15	0.12	0.50	0.05	0.21	0.79
Annual	1.29 ± 0.82	3.36 ± 3.28	0.34 ± 0.31	0.21 ± 0.24	0.15	0.91	0.06	0.34	0.57

Concentrations presented in [μg m^−3^]

^a^Ass-sea salt aerosols (from Eq. 1)

^b^Non-sea salt sulfates (from Eq. 2)

^c^Non-sea salt calcium (from Eq. 3)

^d^Non-sea salt potassium (from Eq. 4)

^e^Period between April and September 2009

^f^Period between January and March 2009 and between October and December 2009

In spring, there were also isolated cases in which high carbon concentrations resulted from traffic related emissions. Such occurrences were most clearly noted at wind speed close to 1 m s^−1^ and manifested themselves in a decrease in the OC/EC ratio value, sometimes by as much as a half in comparison with the annual mean (5.8). For instance, on 3 April 2009, when air masses drifted over Diabla Gora from the SSE (from over Elk) and the average wind speed amounted to 1.2 m s^−1^, a recorded high concentration of EC—in terms of the whole year—was observed in both aerosol fractions (Fig. 6b). The OC/EC ratio at this time dropped to 2.7 (Fig. 3), indicating carbon emission from gas-fuelled transportation (Schauer et al. 2002). Moreover, the NO_3_^−^/SO_4_^2−^ ratio was also high (3.3 and 3.2 for PM2.5 and PM10, respectively), and its increase, in comparison with the annual mean (Table 3), points to the emission of nitrogen compounds from mobile sources (Wang et al. 2005; Lai et al. 2007). In addition to the above, the influence of traffic on carbon concentration in aerosols in Diabla Gora was also observed when air masses were transported from the Polish-Russian border at Goldap, located 29 km away from the station (Figs. 1 and 6c), which is crossed daily by about 800 cars and several dozen heavy-load vehicles each weighing up to 3.5 t. About 50 km away from the station in the same direction and therefore on the Russian side runs the European-grade Kaliningrad-Vilnius-Moscow road (Fig. 1). Northern advection from the abovementioned areas was characterized by concentrations of EC (1.7 and 1.9 in PM2.5 and PM10, respectively) which were three times higher than the annual average and the NO_3_^−^/SO_4_^2−^ ratio higher than 1 (2.2 and 2.0 for PM2.5 and PM10, respectively).

In summer, different sources of secondary and primary OC were noted in PM10 aerosols (*r* = 0.33) with concentrations of OC_prim_ and EC being three times lower than in winter (Fig. 5) as a result of reduced impact of emissions in the community sector, a very high sum of precipitation, and a rather low average wind speed (under 2 m s^−1^) (Table 1). That led to regional emission sources having a limited role in the formation of high concentrations of primary origin carbon, while SOC was found to be predominant both in PM2.5 and PM10, on average accounting for 70 % of the total aerosol mass, and manifested itself in the highest mean of OC/EC ratio values (summer average 11.5) (Fig. 3). Taking into account the location of the measurement station, the high proportion of SOC may be attributed to the emission of volatile organic compounds from plant essential oils and pollens. In addition to this, a rise in temperature over 10 °C (on average 15.7 °C) and intense solar radiation (>50 W m^−2^) were conducive to the oxidation of volatile organic compounds of biological origin, which in turn were transformed into secondary aerosols rich in organic carbon (SOC) (Sánchez de la Campa et al. 2009). Similar observations were made in the summer period by Aurela et al. (2011) at a forest background station in Hyytiälä, Finland.

**Fig. 7:**
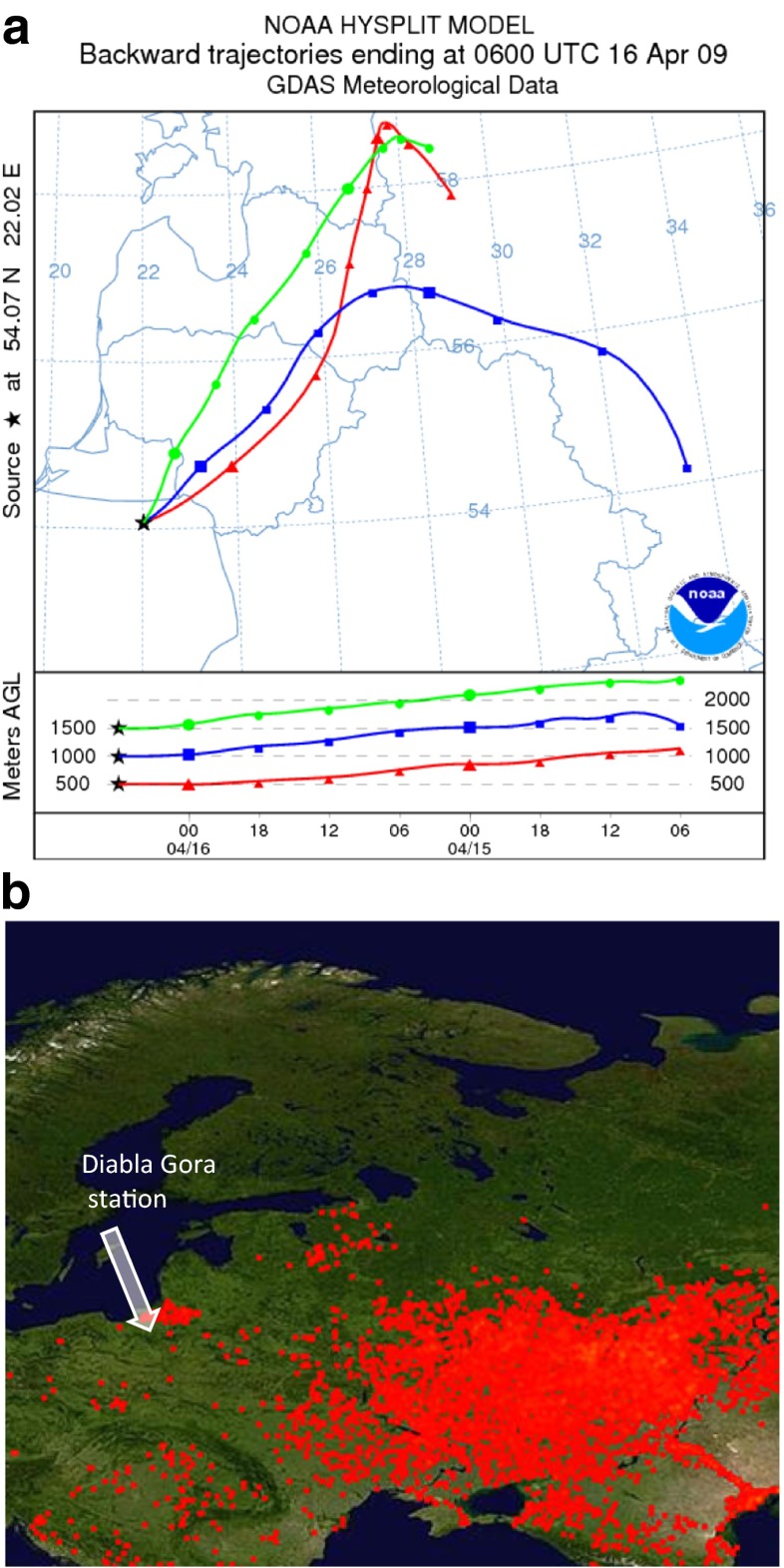
Back trajectories calculated using the HYSPLIT model for Diabla Gora at an altitude of 500 (*triangles*), 1000 (*squares*), and 1500 (*circles*) m a.g.l., ending at 6 UTC on 16 April 2009 (**a**) and fire map for 16 April 2009 (www.rapidfire.sci.gsfc.nasa.gov/) (**b**)

In order to ascertain what part of carbon in aerosols was derived from biomass burning, useful ratios such as nssK^+^/OC and nssK^+^/EC were taken into account, as potassium compounds form aerosols onto which gases from combustion processes become condensed. In other dominant sources of OC and EC, though, such as fossil fuel and vehicle emissions, K^+^ concentration is generally insignificant (Satsangi et al. 2012; Mkoma et al. 2013; Pachauri et al. 2013). In the aerosols of the study area, potassium occurred mainly in the form of nssK^+^ (83.9 % of total K^+^ in PM2.5 and 89.9 % of total K^+^ in PM10), pointing to biomass combustion as a potential source (Cachier et al. 1991), and the obtained annual mean value for the nssK^+^/EC ratio amounted to 0.21 in PM2.5 and 0.34 in PM10 (Table 3), both values falling within the range typical for biomass burning (0.21–0.69) (Satsangi et al. 2012). During spring and summer, the nssK^+^/EC ratio in the PM10 aerosols over Diabla Gora was higher than the annual value indicating burning of agricultural waste as the dominant source of primary carbon. In PM2.5, ratios were much lower but still typical for biomass burning, and the nssK^+^/OC ratios also suggest this to be the main source (Table 3) (Mkoma et al. 2013). Occasionally in the spring of 2009, high carbon species concentrations in PM10 over Diabla Gora were the result of pollutants being transported from more remote areas. At the end of March and for the whole of April, for example, the Kaliningrad Oblast and Lithuania, particularly in the vicinity of Vilnius (Figs. 1 and 7), suffered extensive grass burning and fires (www.rapidfire.sci.gsfc.nasa.gov/). The OC_prim_ concentration in PM10 recorded at that time in Diabla Gora was over four times higher than the average for the entire spring (10.5 μg m^−3^) and the nssK^+^ concentration was the highest for the entire year at 464.5 μg m^−3^ (Table 3). Ratios of nssK^+^/EC and nssK^+^/OC in PM10 equal to 0.58 and 0.05 were also typical for grass burning (Satsangi et al. 2012). This situation was accompanied by the lowest cleaning processes of air in Diabla Gora, the sum of precipitation, and relative air humidity in April being at their lowest for the whole year (Table 1).

During the summer period, the important source of coarse primary aerosols (PM10) was emission from the surface of the soil, when the nssK^+^/EC ratio value was then the highest of the year (0.86, Table 3). It should also be pointed out that summer witnesses plenty of recreational activity on the numerous lakes in the area (over 3000 lakes), including the building of bonfires.

During winter and autumn, in keeping with our former observations, the dominant source as demonstrated by low coefficients of nssK^+^/EC and nssK^+^/OC (Table 3) was coal combustion.

## Conclusions

This paper reports the results of long-term measurements of PM2.5 and PM10 collected in the rural area of Puszcza Borecka (Polish National Nature Reserve). The seasonal and daily variability of EC and primary and secondary organic carbon concentrations and their contribution in PM2.5 and PM10 were determined under the influence of the changeable weather conditions. The study contributes to a better understanding of source apportionment of carbon fractions in PM2.5 and PM10 aerosols in the central-eastern part of Europe.

The obtained results made it possible to indicate a significant share of carbon compounds in both PM2.5 (34.5 % of total mass) and PM10 (27.5 % of total mass), with the concentrations of all carbon fractions in PM2.5 diminishing from season to season in the following order: winter > spring > autumn > summer. In PM10, such a tendency was only observed for SOC, while OC_prim_ and EC concentrations decreased as follows: spring > winter > autumn > summer.

In the non-heating period, when emissions from urban sources were limited, carbon compounds were found to originate mainly from agriculture and biomass burning, although secondary and primary organic carbon in PM10 aerosols demonstrated different sources of origin. In spring and autumn, the high concentration of primary organic carbon determined in aerosols, combined with ammonium nitrate and raised concentrations of calcium and potassium, was related to agricultural activity and emission from surrounding fields enriched with mineral elements and fertilizers. This was most clearly defined in PM10. The increase in primary carbon fraction observed in spring was also the result of biomass burning, especially of agricultural waste and from fires in Lithuania and in the Kaliningrad Oblast, and the process became intensified with decreased relative humidity and precipitation together with increased temperature. In summertime, the proportion of SOC rose to 70 % of total PM2.5 and PM10 aerosol mass as a consequence of increased vegetation, a rise in temperature, and intense solar radiation. OC_prim_ and EC were then characterized by the lowest concentrations due to reduced emissions from the community sector and the highest levels of precipitation.

The increase of carbon concentration in winter resulted from low temperatures, low boundary layer, and weak dispersion of pollutants at low wind speed (not exceeding 2.5 m s^−1^) with air masses drifting from over nearby habitations. Local and regional emission sources, within a radius of 50 km from the station, played an important role in the shaping of SOC, OC_prim_, and EC concentrations as, at such times, organic and EC had a shared source in domestic heating. Traffic played a smaller role although high concentrations, particularly of elemental and primary organic carbon and nitrates, were noted in pollutants transported from over nearby cities and the Polish-Russian border, located 29 km away from the station.
